# The subjective knee value is a valid single-item survey to assess knee function in common knee disorders

**DOI:** 10.1007/s00402-021-03794-3

**Published:** 2021-02-01

**Authors:** Fabian Plachel, Tobias Jung, Benjamin Bartek, Katja Rüttershoff, Carsten Perka, Clemens Gwinner

**Affiliations:** Center for Musculoskeletal Surgery, Campus Mitte, Charité–Universitaetsmedizin Berlin, Charitéplatz 1, 10117 Berlin, Germany

**Keywords:** PROM, Subjective knee value, Knee function, Knee disorder, Evaluation

## Abstract

**Introduction:**

The patient’s perspective plays a key role in judging the effect of knee disorders on physical function. We have introduced the Subjective Knee Value (SKV) to simplify the evaluation of individual’s knee function by providing one simple question. The purpose of this prospective study was to validate the SKV with accepted multiple-item knee surveys across patients with orthopaedic knee disorders.

**Materials and methods:**

Between January through March 2020, consecutive patients (*n* = 160; mean age 51 ± 18 years, range from 18 to 85 years, 54% women) attending the outpatient clinic for knee complaints caused by osteoarthritis (*n* = 69), meniscal lesion (*n* = 45), tear of the anterior cruciate ligament (*n* = 23) and focal chondral defect (*n* = 23) were invited to complete a knee-specific survey including the SKV along with the Knee Injury Osteoarthritis Outcome Score (KOOS) and the International Knee Documentation Committee subjective knee form (IKDC-S). The Pearson correlation coefficient was used to evaluate external validity between the SKV and each patient-reported outcome measure (PROM) separately. Furthermore, patient’s compliance was assessed by comparing responding rates.

**Results:**

Overall, the SKV highly correlated with both the KOOS (*R* = 0.758, *p* < 0.05) and the IKDC-S (*R* = 0.802, *p* < 0.05). This was also demonstrated across all investigated diagnosis- and demographic-specific (gender, age) subgroups (range 0.509–0.936). No relevant floor/ceiling effects were noticed. The responding rate for the SKV (96%) was significantly higher when compared with those for the KOOS (81%) and the IKDC-S (83%) (*p* < 0.05).

**Conclusion:**

At baseline, the SKV exhibits acceptable validity across all investigated knee-specific PROMs in a broad patient population with a wide array of knee disorders**.** The simplified survey format without compromising the precision to evaluate individual’s knee function justifies implementation in daily clinical practice.

**Level of evidence:**

II, cohort study (diagnosis).

## Introduction

Traumatic injuries and degenerative changes of the knee joint are the most common cause of disability among all ages, causing reduced quality of life (QoL) [1, 2]. Therefore, improving patient’s QoL and symptoms are key objectives for surgery to treat knee disorders [3–8]. As a result, numerous injury-specific rating scales have been utilized in an attempt to enable the assessment of patient’s health status, associated knee impairments and treatment effects [9].

Although physician-based knee surveys have been routinely used in the past decades [10, 11], the patient’s perspective has become increasingly important in recent years. Of note, patient-reported outcome measures (PROMs) have become valid instruments, which are completed by the patient without interpretation of the patient’s response by a physician or anyone else [12]. Among a core outcome measure set, the Knee Injury Osteoarthritis Outcome Score (KOOS) [13] and the International Knee Documentation Committee (IKDC) [14] knee survey are validated multiple-item tools for comprehensive assessment of knee disorders in both clinical and research contexts.

However, the implementation of those complex PROMs in daily practice is often fraught with problems. As survey length and complexity negatively affect patient’s compliance and response rate, the validity of research data is limited by excluding a substantial proportion of the study sample [15–17]. Thus, single-item measures were evolved to reduce response and administrative burden [18–20]. Beyond them, the Subjective Shoulder Value (SSV) [18], the Subjective Elbow Value (SEV) [19] and the Single Assessment Numeric Evaluation (SANE) [21] survey were previously developed as efficient single-item scores for assessing the functional status of the upper extremity. It is widely accepted that those rating scales are responsive, valid and effective in a wide array of pathologies [19, 20, 22–25].

We have recently introduced the Subjective Knee Value (SKV) for the knee joint to improve compliance while maintaining the integrity of a validated survey format. The SKV simplifies patient evaluation by providing one simple question. Although some studies demonstrated the efficiency of single-item measures to capture musculoskeletal health status [18, 23, 26–28], the utilization as a knee-specific PROM to a wide spectrum of knee pathologies is largely unknown [22].

The main goal of this prospective study was to validate the SKV in a broad patient population with a wide array of orthopaedic knee disorders. Therefore, it was hypothesized that the SKV provides a higher response rate when compared to accepted joint-specific PROMs, including the KOOS and IKDC subjective knee evaluation form (IKDC-S) [29]. Furthermore, we hypothesized that the SKV was able to discriminate between the affected knee joint and the unaffected side. Finally, it was hypothesized that the SKV correlated with the multiple-question alternatives.

## Methods

Prior to the beginning of this prospective single-center study, approval of the local ethical committee was obtained (EA/030/20). Each patient signed the written informed consent.

All consecutive patients who presented at our outpatient clinic for any knee disorder between January and March 2020 were enrolled. On the day of consultation, each patient completed a standardized self-administered survey, which was provided upon arrival. The survey collected demographic baseline data including age, gender and major complaint for consultation. To determine the SKV, all patients were asked to rate subjectively their current knee function for both the affected and non-affected side. The maximum score is 100% indicating no problem and the minimum score is 0% indicating a severe problem to the knee joint. As originally introduced by Gilbart and colleagues for the shoulder joint [18], all patients had to answer the following modified question: **“What is the overall percent value of your knee joint if a completely normal knee joint represents 100%?”** or in other words: **“A completely normal knee joint would cost 100€. How much would you be willing to pay for your knee joint?”**.

The survey further included the KOOS [13] and the IKDC-S [29] scores. The KOOS measures subjectively the patient’s opinion about their knee joint by providing 42 items across 5 subscales and has been validated for several knee disorders [30–32]. The maximum score is 100 points indicating no knee problems, whereas a score of 0 points indicates extreme knee problems. The IKDC-S was developed to scale subjective knee function in patients with a variety of knee conditions providing a total of 18 items [33–35]. The possible score ranges from 0 to 100 points, whereas 100 points indicate the absence of any symptoms. For both PROMs, the scoring is considered invalid if 2 or more items are missing [9]. The completed survey was collected and evaluated by the treating physician during consultation. Subsequently, the number of missing items was documented for each measure. To provide scoring, missing items were required by the treating physician and answered by the patient. Furthermore, the specific diagnosis was made according to the anamnesis and clinical as well as radiological assessment. Patients with (1) knee osteoarthritis, (2) meniscal tear, (3) lesion of the anterior cruciate ligament (ACL) or (4) focal chondral defect were included. Exclusion criteria were patient age below 18 years and previous surgeries on the affected knee joint.

Statistical analyses were performed with SPSS Statistics 24.0 software (IBM, Armonk, NY, USA). A *p* value less than 0.05 was considered statistically significant. Descriptive statistics (mean, standard deviation, minimum and maximum values, frequency, percentage and floor/ceiling rates) were calculated for all PROMs. The Pearson correlation coefficient (R) with a 95% Confidence Interval (CI) was used to assess the relationship, strength and the direction of the association (i.e. external validity) between the SKV and each PROM (KOOS, IKDC-S) for the entire study population and across diagnosis- and demographic-specific (gender and age categories: 18–30, 30–50, 50–70, > 70 years) subgroups. Correlation strength was defined as very high if the *R* value was above 0.90, as high if the *R* value was between 0.70 and 0.89, moderate if the *R* value was between 0.50 and 0.69, low if the *R* value was between 0.30 and 0.49, and negligible if the *R* value was below 0.30. Bland–Altman plots were used to visualize agreement of two rating scales [36]. Hence, the *x* axis represents the mean and the *y* axis represents the difference of two measurements. For the discriminant analysis, the paired *t* test was used to compare the SKV between the affected and the non-affected knee joint. The ANOVA was determined to evaluate the mean differences among subgroups.

## Results

A total of 160 surveys in 160 patients (74 men, 86 women) with a mean age of 51 ± 18 years (range, from 18 to 85 years) was analyzed. According to the underlying cause for consultation, knee osteoarthritis was found in 69 patients (44%), meniscal lesion in 45 patients (28%), ACL tear in 23 patients (14%) and focal chondral defect in 23 patients (14%). Further baseline demographic data with regards to the specific diagnoses are summarized in Table 1.

**Table 1:** Baseline demographic data and knee measures for the entire study population and with regards to the diagnoses (Group 1 = knee osteoarthritis, Group 2 = meniscal lesion, Group 3 = tear of the anterior cruciate ligament, Group 4 focal chondral defect)

Variables*	Total *N* = 160	Group 1 *N* = 69	Group 2 *N* = 45	Group 3 *N* = 23	Group 4 *N* = 23
Age, years	51 ± 18	67 ± 9	44 ± 14	34 ± 8	33 ± 13
Sex, male:female	74:86	24:45	24:21	16:7	10:13
SKV affected, %	44 ± 24	35 ± 19	48 ± 22	49 ± 28	57 ± 25
Invalid, %	4	6	2	4	3
SKV, non-affected, %	88 ± 20	81 ± 23	94 ± 18	95 ± 11	91 ± 16
Invalid, %	3	5	2	1	3
KOOS, points	46 ± 23	36 ± 17	51 ± 22	52 ± 25	61 ± 24
Invalid, %	19	25	20	17	4
IKDC-S, points	43 ± 19	35 ± 13	47 ± 18	49 ± 23	56 ± 21
Invalid, %	17	15	27	13	9

*Data are reported as mean ± SD; *SKV* subjective knee value, *KOOS* Knee Injury Osteoarthritis Outcome Score, *IKDC-S* International Knee Documentation Committee subjective knee evaluation form

While the SKV was completely missing in only 6 patients (4%), the KOOS was incomplete in 56 patients (35%) and the IKDC-S in 62 patients (39%) (*p* < 0.05). A mean of 3 ± 3 (range, from 1 to 7) items were missing for the KOOS and 2 ± 1 (range, from 1 to 5) for the IKDC-S. In terms of interpretation (invalid if 2 or more items were missing), the KOOS was initially considered invalid in 31 patients (19%) and the IKDC-S in 27 patients (17%) (Table 1). The rate of valid responses were significantly different among the PROMs (*p* < 0.05). Floor and ceiling effects were comparable across all PROMs (SKV: 2%-1%; KOOS: 1%-1%; IKDC-S: 1%-0%).

Table 1 further demonstrates the mean diagnosis-specific PROMs. Irrespectively, the mean SKV was significantly different between the affected knee joint and the non-affected knee joint (*p* < 0.05). Both patient age (*R* = − 0.304, *p* < 0.05) and gender (male, 91% ± 16% vs. female, 85% ± 23%; *p* < 0.05) significantly affected the mean SKV of the healthy knee joint.

Overall, the SKV had a high positive correlation to the KOOS (*R* = 0.758, 95% CI 0.655–0.860; *p* < 0.05) and the IKDC-S (*R* = 0.802, 95% CI 0.708–0.896; *p* < 0.05) (Table 2). Interestingly, the relationship between the KOOS and the IKDC-S was very high (*R* = 0.918, 95% CI 0.856–0.980) as well. Further results demonstrating correlations between the PROMs across all diagnosis-specific subgroups are summarized in Table 2.

**Table 2:** Correlations between the SKV and subjective outcome measures with regard to the diagnoses

Correlations	*R*	95% CI		Relationship
		Lower bound	Upper bound	
Overall, *N* = 160				
SKV vs. KOOS	0.758	0.655	0.860	High
SKV vs. IKDC-S	0.802	0.708	0.896	High
KOOS vs. IKDC-S	0.918	0.856	0.980	Very high
Knee osteoarthritis, *N* = 69				
SKV vs. KOOS	0.509	0.321	0.771	Moderate
SKV vs. IKDC-S	0.593	0.460	0.916	Moderate
KOOS vs. IKDC-S	0.850	0.781	1.059	High
Meniscal lesion, *N* = 45				
SKV vs. KOOS	0.789	0.583	0.950	High
SKV vs. IKDC-S	0.779	0.574	0.952	High
KOOS vs. IKDC-S	0.915	0.798	1.048	Very high
ACL tear, *N* = 23				
SKV vs. KOOS	0.817	0.597	1.159	High
SKV vs. IKDC-S	0.881	0.655	1.077	High
KOOS vs. IKDC-S	0.925	0.689	1.004	Very high
Focal chondral defect, *N* = 23				
SKV vs. KOOS	0.862	0.629	1.087	High
SKV vs. IKDC-S	0.923	0.700	1.029	Very high
KOOS vs. IKDC-S	0.957	0.778	1.024	Very high

*R* correlation coefficient, *CI* confidence interval, *SKV* subjective knee value, *KOOS* Knee Injury Osteoarthritis Outcome Score, *IKDC-S* International Knee Documentation Committee subjective knee evaluation form

In the subset analyses for gender and patient age, there were only minimal differences in correlations (Table 3). Only relationship between the PROMs in patients above the age of 70 years deviated to some degree.

**Table 3:** Correlations between the SKV and subjective outcome measures with regard to sex and patient age

Correlations	*R*	95% CI		Relationship
		Lower bound	Upper bound	
Sex, male				
SKV vs. KOOS	0.819	0.688	0.959	High
SKV vs. IKDC-S	0.858	0.717	0.951	High
KOOS vs. IKDC-S	0.936	0.824	0.984	Very high
Sex, female				
SKV vs. KOOS	0.746	0.599	0.840	High
SKV vs. IKDC-S	0.710	0.583	0.919	High
KOOS vs. IKDC-S	0.878	0.806	1.023	High
Age group, 18–30 years				
SKV vs. KOOS	0.714	0.395	0.951	High
SKV vs. IKDC-S	0.812	0.462	0.865	High
KOOS vs. IKDC-S	0.894	0.612	0.939	High
Age group, 30–50 years				
SKV vs. KOOS	0.757	0.578	0.983	High
SKV vs. IKDC-S	0.829	0.667	1.006	High
KOOS vs. IKDC-S	0.916	0.778	1.014	Very high
Age group, 50–70 years				
SKV vs. KOOS	0.716	0.511	0.873	High
SKV vs. IKDC-S	0.683	0.510	0.920	Moderate
KOOS vs. IKDC-S	0.911	0.866	1.106	Very high
Age group, > 70 years				
SKV vs. KOOS	0.511	0.122	0.864	Moderate
SKV vs. IKDC-S	0.596	0.342	1.120	Moderate
KOOS vs. IKDC-S	0.779	0.582	1.124	High

*R* correlation coefficient, *CI* confidence interval, *SKV* subjective knee value, *KOOS* Knee Injury Osteoarthritis Outcome Score, *IKDC-S* International Knee Documentation Committee subjective knee evaluation form

**Fig. 1:**
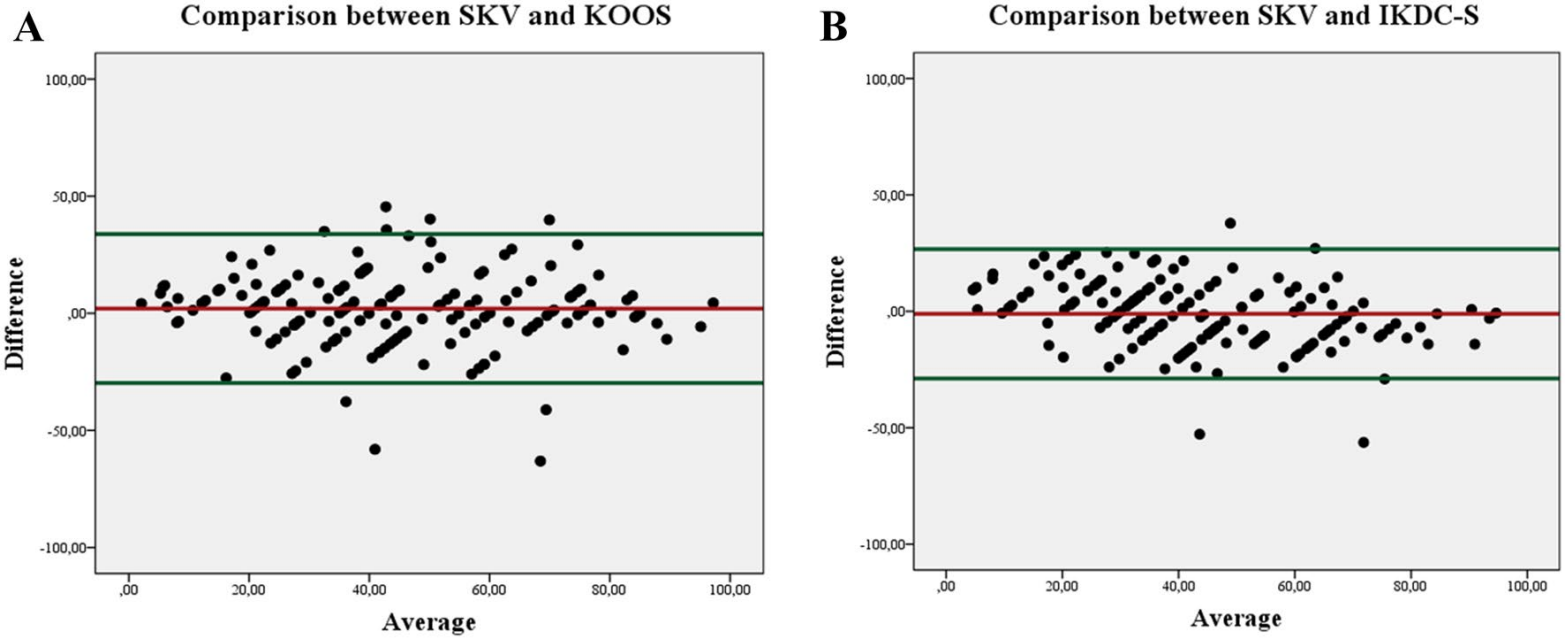
Bland–Altman analysis scatterplots for the precision of agreement (green lines; ± 1.96 times the standard deviation around the bias) are shown for the comparison of the Subjective Knee Value (SKV) to **a** the Knee Injury Osteoarthritis Outcome Score (KOOS) and **b** the IKDC-S = International Knee Documentation Committee subjective knee evaluation form. The red line indicates the bias (SKV vs. KOOS: 2.02; SKV vs. IKDC: − 1.08)

Figure 1 demonstrates the precision of agreement for two sets of measurements (A: SKV versus KOOS, 94%; B: SKV versus IKDC-S, 98%). The majority of the measure differences is within the 95% limits of agreement indicating that the SKV does not affect clinical interpretation of the data.

## Discussion

The main finding of the current study was that the SKV exhibits acceptable validity across all investigated knee-specific PROMs at baseline. The results further suggest its utilization in a broad patient population with a wide array of knee disorders. Besides that, the responding rate above 95% indicates its simplicity to assess knee function. Furthermore, the SKV was able to detect significant differences between the affected knee joint and the healthy contralateral side. Hence, all hypotheses were confirmed.

In general, self-administered PROMs are of great importance in clinical decision-making and clinical research encouraging a patient-focused approach. It is highly recommended to keep outcome measures short and simple ensuring data quality while maximizing patient’s compliance and minimizing response burden, especially for those for which long questionnaires may be onerous. This key concept provided the basis for introducing single-item surveys (e.g. SANE score [20] developed in the US or SSV [18] as well as SEV [19] developed in Europe) which were originally used for disorders of the upper extremity [18–20, 23, 25]. Given its ease to use, the SANE scale was subsequently validated and utilized for patients with knee ligament injury [21, 26, 28] or knee osteoarthritis [27, 37, 38], demonstrating moderate correlations (mean 0.60 ± 0.24, range from 0.12 to 0.88) to knee-specific multiple-item PROMs [24]. However, some major concerns with current literature exist. First of all, the lack of standardization of the measurement tools for knee disorders makes study comparison difficult at best and impossible to draw accurate conclusions. For example, the Lysholm rating scale [11] was used to validate single-item surveys in patients with either ligamentous lesions [21] or osteoarthritic changes [37] of the knee joint. However, van Meer and colleagues recently demonstrated that the IKDC-S rather than the Lysholm scale is the most useful injury-specific PROM to evaluate patients with ACL tears [39]. In that specific group, a strong correlation between the IKDC-S and the SKV was demonstrated in the current study. Furthermore, the IKDC-S provided a score with best measurement properties in patients with meniscal injury [40]. To the best of our knowledge, we were the first to validate a single-item survey in patients suffering knee complaints caused by meniscal lesion. Besides that, the KOOS [13] provides the most persuasive evidence of efficacy to assess both early-onset osteoarthritis including focal chondral lesion and more severe arthritic changes of the knee joint [30, 41]. To date, no study analyzed the agreement between a single-item PROM and the full-version KOOS. In the present study, a high correlation between the SKV and the KOOS was found.

Given the inherently problematic nature of survey complexity, it is preferable to apply shorter instruments. For example, the KOOS [13] includes a plethora of items inquiring about activity-related pain, daily knee symptoms, complaints associated with sport activities and QoL. Consequently, missing data are common with reported rates ranging from 0.8% to 74.0% in patients who underwent knee arthroscopy or total knee replacement, respectively [30, 42]. In line with previous studies, it was found that missing items (e.g. stated as not applicable) were primarily reported on activity-related subscales. Furthermore, low scores may not stringently indicate poor knee function but rather be due to general poor health or other illness [43–45]. In the current study, a group of patients with knee osteoarthritis mentioned severe problems while running caused by chronic obstructive pulmonary disease or cardiac issues rather than the knee joint itself. Notably, some items were difficult to complete for older patients without guidance. As a consequence, misunderstanding or misinterpretation further led to slight disagreement between the SKV and both selected PROMs in the elderly (Table 3). Similar to the KOOS, 10% of the patients in the study by Irrgang and colleagues [29] and 17% of our study population did not complete the entire IKDC-S questionnaire, impairing the representativeness of results.

The main strength of the presented study is that the data was collected in a large study cohort and in a prospective fashion. Furthermore, to unravel the persistent inconsistency regarding the PROM of first choice, we secondarily evaluated the agreement of two widely accepted and highly used knee-specific measures. According to the results, a minimum data set including one multiple-question PROM together with the SKV offers an effective opportunity to improve measurement precision and to ease implementation into the clinical workflow while reducing respondent and collection burden. The SKV can be administered to the entire study population as an adjunction to traditional outcome measures. In line with previous studies, we highly recommend to use the KOOS in patients with cartilaginous or osteoarthritic changes and the IKDC-S in patients with either ligamentous or meniscal lesions of the knee joint.

However, our study is limited by the fact that not all quality properties for PROMs were evaluated. Although external validity and interpretability were confirmed for the SKV, other properties such as reliability (test–retest; inter- and intraobserver reliability) and responsiveness were not yet evaluated and should be investigated in further studies. Furthermore, we did not answer the question if the SKV is able to detect either improvement or worsening of individual’s knee function after treatment (e.g. arthroscopic surgery or total knee replacement). Nevertheless, Shelbourne and colleagues found a strong correlation between a single-item survey and the IKDC-S for all ages and both sexes in more than 10.000 cases after ACL repair and knee arthroscopy [26]. As no reference data (assessed in a healthy population) for single-item scales are available in literature, our study may help to individually adjust treatment effects by providing mean values in a non-affected knee joint. Similar to the studies by Paradowski et al. and Marot et al. evaluating reference values for the KOOS [46, 47], a significant decrease of the SKV was found with increased patient age and in females. Finally, the SKV does not allow to inquire the main reason for consultation (e.g. knee pain, function, or stability). However, this might be elicited by providing one more simple question.

## Conclusions

At baseline, the Subjective Knee Value exhibits acceptable validity across all investigated knee-specific PROMs in a broad patient population with a wide array of knee disorders**.** The simplified survey format without compromising the precision to evaluate individual’s knee function justifies implementation in daily clinical practice.

## References

[1] Filbay SR, Culvenor AG, Ackerman IN, Russell TG, Crossley KM (2015). Quality of life in anterior cruciate ligament-deficient individuals: a systematic review and meta-analysis. Br J Sports Med.

[2] Törmälehto S, Mononen ME, Aarnio E, Arokoski JPA, Korhonen RK, Martikainen J (2018). Health-related quality of life in relation to symptomatic and radiographic definitions of knee osteoarthritis: data from osteoarthritis initiative (OAI) 4-year follow-up study. Health Qual Life Outcomes.

[3] Goh GS, Abd Razak HRB, Tay DKJ, Lo NN, Yeo SJ (2020). Early post-operative oxford knee score and knee society score predict patient satisfaction 2 years after total knee arthroplasty. Arch Orthop Trauma Surg.

[4] Konopka JF, Lee Y-Y, Su EP, McLawhorn AS (2018). Quality-adjusted life years after hip and knee arthroplasty: health-related quality of life after 12,782 joint replacements. JB JS Open Access.

[5] Filbay SR, Ackerman IN, Russell TG, Macri EM, Crossley KM (2014). Health-related quality of life after anterior cruciate ligament reconstruction: a systematic review. Am J Sports Med.

[6] Costa-Paz M, Garcia-Mansilla I, Marciano S, Ayerza MA, Muscolo DL (2019). Knee-related quality of life, functional results and osteoarthritis at a minimum of 20 years’ follow-up after anterior cruciate ligament reconstruction. Knee.

[7] Thaunat M, Fournier G, O’Loughlin P, Kouevidjin BT, Clowez G, Borella M (2020). Clinical outcome and failure analysis of medial meniscus bucket-handle tear repair: a series of 96 patients with a minimum 2 year follow-up. Arch Orthop Trauma Surg.

[8] Kaiser N, Jakob RP, Pagenstert G, Tannast M, Petek D (2020). Stable clinical long term results after AMIC in the aligned knee. Arch Orthop Trauma Surg.

[9] Collins NJ, Misra D, Felson DT, Crossley KM, Roos EM (2011). Measures of knee function: International Knee Documentation Committee (IKDC) Subjective Knee Evaluation Form, Knee Injury and Osteoarthritis Outcome Score (KOOS), Knee Injury and Osteoarthritis Outcome Score Physical Function Short Form (KOOS-PS), Knee Outcome Survey Activities of Daily Living Scale (KOS-ADL), Lysholm Knee Scoring Scale, Oxford Knee Score (OKS), Western Ontario and McMaster. Arthritis Care Res.

[10] Müller W, Biedert R, Hefti F, Jakob RP, Munzinger U, Stäubli HU (1988) OAK knee evaluation. A new way to assess knee ligament injuries. Clin Orthop Relat Res p. 37–50 3383501

[11] Lysholm J, Gillquist J (1982). Evaluation of knee ligament surgery results with special emphasis on use of a scoring scale. Am J Sports Med.

[12] Hildon Z, Neuburger J, Allwood D, Van Der Meulen J, Black N (2012). Clinicians and patients views of metrics of change derived from patient reported outcome measures (PROMs) for comparing providers performance of surgery. BMC Health Serv Res.

[13] Roos EM, Roos HP, Lohmander LS, Ekdahl C, Beynnon BD (1998). Knee injury and osteoarthritis outcome score (KOOS)—development of a self-administered outcome measure. J Orthop Sports Phys Ther.

[14] Hefti E, Müller W, Jakob RP, Stäubli HU (1993). Evaluation of knee ligament injuries with the IKDC form. Knee Surg Sports Traumatol Arthrosc.

[15] Rombach I, Rivero-Arias O, Gray AM, Jenkinson C, Burke Ó (2016). The current practice of handling and reporting missing outcome data in eight widely used PROMs in RCT publications: a review of the current literature. Qual Life Res.

[16] Gomes M, Gutacker N, Bojke C, Street A (2016). Addressing missing data in patient-reported outcome measures (PROMS): implications for the use of PROMS for comparing provider performance. Health Econ.

[17] Perneger TV, Chamot E, Bovier PA (2005). Nonresponse bias in a survey of patient perceptions of hospital care. Med Care.

[18] Gilbart MK, Gerber C (2007). Comparison of the subjective shoulder value and the constant score. J Shoulder Elbow Surg.

[19] Schneeberger AG, Kösters MC, Steens W (2014). Comparison of the subjective elbow value and the mayo elbow performance score. J Shoulder Elbow Surg.

[20] Williams GN, Gangel TJ, Arciero RA, Uhorchak JM, Taylor DC (1999). Comparison of the single assessment numeric evaluation method and two shoulder rating scales. Outcomes measures after shoulder surgery. Am J Sports Med.

[21] Williams GN, Taylor DC, Gangel TJ, Uhorchak JM, Arciero RA (2000). Comparison of the single assessment numeric evaluation method and the Lysholm score. Clin Orthop Relat Res.

[22] Garcia AN, Cook C, Lutz A, Thigpen CA (2019). Concurrent validity of the single assessment numerical evaluation and patient-reported functional measures in patients with musculoskeletal disorders: an observational study. Musculoskelet Sci Pract.

[23] Razaeian S, Wiese B, Zhang D, Krettek C, Meller R, Hawi N (2020). Correlation between Oxford elbow score and single assessment numeric evaluation: is one simple question enough?. J Shoulder Elbow Surg.

[24] O’Connor CM, Ring D (2019). Correlation of single assessment numeric evaluation (SANE) with other patient reported outcome measures (PROMs). Arch Bone Jt Surg.

[25] Thigpen CA, Shanley E, Momaya AM, Kissenberth MJ, Tolan SJ, Tokish JM (2018). Validity and responsiveness of the single alpha-numeric evaluation for shoulder patients. Am J Sports Med.

[26] Shelbourne KD, Barnes AF, Gray T (2012). Correlation of a single assessment numeric evaluation (SANE) rating with modified Cincinnati knee rating system and IKDC subjective total scores for patients after ACL reconstruction or knee arthroscopy. Am J Sports Med.

[27] Austin DC, Torchia MT, Werth PM, Lucas AP, Moschetti WE, Jevsevar DS (2019). A one-question patient-reported outcome measure is comparable to multiple-question measures in total knee arthroplasty patients. J Arthroplasty.

[28] Winterstein AP, McGuine TA, Carr KE, Hetzel SJ (2013). Comparison of IKDC and SANE outcome measures following knee injury in active female patients. Sports Health.

[29] Irrgang JJ, Anderson AF, Boland AL, Harner CD, Kurosaka M, Neyret P (2001). Development and validation of the International Knee Documentation Committee Subjective Knee Form. Am J Sports Med.

[30] Roos EM, Toksvig-Larsen S (2003). Knee injury and Osteoarthritis Outcome Score (KOOS)—validation and comparison to the WOMAC in total knee replacement. Health Qual Life Outcomes.

[31] Salavati M, Akhbari B, Mohammadi F, Mazaheri M, Khorrami M (2011). Knee injury and Osteoarthritis Outcome Score (KOOS); reliability and validity in competitive athletes after anterior cruciate ligament reconstruction. Osteoarthritis Cartil.

[32] Bekkers JEJ, de Windt TS, Raijmakers NJH, Dhert WJA, Saris DBF (2009). Validation of the Knee Injury and Osteoarthritis Outcome Score (KOOS) for the treatment of focal cartilage lesions. Osteoarthritis Cartil.

[33] Agel J, Laprade RF (2009). Assessment of differences between the modified Cincinnati and International Knee Documentation Committee patient outcome scores: a prospective study. Am J Sports Med.

[34] Crawford K, Briggs KK, Rodkey WG, Steadman JR (2007). Reliability, validity, and responsiveness of the IKDC score for meniscus injuries of the knee. Arthroscopy.

[35] Greco NJ, Anderson AF, Mann BJ, Cole BJ, Farr J, Nissen CW (2010). Responsiveness of the international knee documentation committee subjective knee form in comparison to the western Ontario and McMaster universities osteoarthritis index, modified Cincinnati knee rating system, and short form 36 in patients with focal articular cartilage defects. Am J Sports Med.

[36] Bland JM, Altman DG (1999). Measuring agreement in method comparison studies. Stat Methods Med Res.

[37] Sueyoshi T, Emoto G, Yato T (2018). Correlation between Single assessment numerical evaluation score and Lysholm score in primary total knee arthroplasty patients. Arthroplast Today.

[38] Pietrosimone B, Luc BA, Duncan A, Saliba SA, Hart JM, Ingersoll CD (2017). Association between the single assessment numeric evaluation and the Western Ontario and McMaster Universities Osteoarthritis Index. J Athl Train.

[39] Van Meer BL, Meuffels DE, Vissers MM, Bierma-Zeinstra SMA, Verhaar JAN, Terwee CB (2013). Knee injury and osteoarthritis outcome score or international knee documentation committee subjective knee form: which questionnaire is most useful to monitor patients with an anterior cruciate ligament rupture in the short term?. Arthrosc J Arthrosc Relat Surg.

[40] van de Graaf VA, Wolterbeek N, Scholtes VAB, Mutsaerts ELAR, Poolman RW (2014). Reliability and validity of the IKDC, KOOS, and WOMAC for patients with meniscal injuries. Am J Sports Med.

[41] Engelhart L, Nelson L, Lewis S, Mordin M, Demuro-Mercon C, Uddin S (2012). Validation of the knee injury and osteoarthritis outcome score subscales for patients with articular cartilage lesions of the knee. Am J Sports Med.

[42] Roos EM, Roos HP, Ekdahl C, Lohmander LS (2007). Knee injury and Osteoarthritis Outcome Score (KOOS)—validation of a Swedish version. Scand J Med Sci Sports.

[43] Jeong J-N, Kim S-H, Park K-N (2019). Relationship between objectively measured lifestyle factors and health factors in patients with knee osteoarthritis: the STROBE study. Medicine (Baltimore).

[44] Eitner A, Culvenor AG, Wirth W, Schaible H, Eckstein F (2020). Impact of diabetes mellitus on knee osteoarthritis pain and physical and mental status: data from the osteoarthritis initiative. Arthritis Care Res (Hoboken).

[45] Jacobs CA, Vranceanu AM, Thompson KL, Lattermann C (2020). Rapid progression of knee pain and osteoarthritis biomarkers greatest for patients with combined obesity and depression: data from the osteoarthritis initiative. Cartilage.

[46] Paradowski PT, Bergman S, Sundén-Lundius A, Lohmander LS, Roos EM (2006). Knee complaints vary with age and gender in the adult population. Population-based reference data for the knee injury and osteoarthritis outcome score (KOOS). BMC Musculoskelet Disord.

[47] Marot V, Murgier J, Carrozzo A, Reina N, Monaco E, Chiron P (2019). Determination of normal KOOS and WOMAC values in a healthy population. Knee Surg Sports Traumatol Arthrosc.

